# Comparative performance of large language models in structuring head CT radiology reports: multi-institutional validation study in Japan

**DOI:** 10.1007/s11604-025-01799-1

**Published:** 2025-05-14

**Authors:** Hirotaka Takita, Shannon L. Walston, Yasuhito Mitsuyama, Ko Watanabe, Shoya Ishimaru, Daiju Ueda

**Affiliations:** 1 Department of Diagnostic and Interventional Radiology, Graduate School of Medicine, Osaka Metropolitan University, 1-4-3 Asahi-Machi, Abeno-ku, Osaka, 545-8585 Japan; 2 Department of Artificial Intelligence, Graduate School of Medicine, Osaka Metropolitan University, 1-4-3 Asahi-Machi, Abeno-ku, Osaka, 545-8585 Japan; 3 Smart Data and Knowledge Services Department, German Research Center for Artificial Intelligence (DFKI GmbH), 67663 Kaiserslautern, Germany; 4 Department of Core Informatics, Graduate School of Informatics, Osaka Metropolitan University, 1-1, Gakuen-cho, Naka-ku, Sakai, 599-8531 Japan

**Keywords:** Large language model, Free-text radiology report, Structured radiology report, Japan medical imaging database, Intracranial hemorrhage, Skull fracture

## Abstract

**Purpose:**

To compare the diagnostic performance of three proprietary large language models (LLMs)—Claude, GPT, and Gemini—in structuring free-text Japanese radiology reports for intracranial hemorrhage and skull fractures, and to assess the impact of three different prompting approaches on model accuracy.

**Materials and methods:**

In this retrospective study, head CT reports from the Japan Medical Imaging Database between 2018 and 2023 were collected. Two board-certified radiologists established the ground truth regarding intracranial hemorrhage and skull fractures through independent review and consensus. Each radiology report was analyzed by three LLMs using three prompting strategies—Standard, Chain of Thought, and Self Consistency prompting. Diagnostic performance (accuracy, precision, recall, and F1-score) was calculated for each LLM–prompt combination and compared using McNemar’s tests with Bonferroni correction. Misclassified cases underwent qualitative error analysis.

**Results:**

A total of 3949 head CT reports from 3949 patients (mean age 59 ± 25 years, 56.2% male) were enrolled. Across all institutions, 856 patients (21.6%) had intracranial hemorrhage and 264 patients (6.6%) had skull fractures. All nine LLM–prompt combinations achieved very high accuracy. Claude demonstrated significantly higher accuracy for intracranial hemorrhage than GPT and Gemini, and also outperformed Gemini for skull fractures (*p* < 0.0001). Gemini’s performance improved notably with Chain of Thought prompting. Error analysis revealed common challenges including ambiguous phrases and findings unrelated to intracranial hemorrhage or skull fractures, underscoring the importance of careful prompt design.

**Conclusion:**

All three proprietary LLMs exhibited strong performance in structuring free-text head CT reports for intracranial hemorrhage and skull fractures. While the choice of prompting method influenced accuracy, all models demonstrated robust potential for clinical and research applications. Future work should refine the prompts and validate these approaches in prospective, multilingual settings.

**Supplementary Information:**

The online version contains supplementary material available at 10.1007/s11604-025-01799-1.

## Introduction

Intracranial hemorrhage and skull fractures are critical acute findings in head trauma, often requiring prompt diagnosis to guide urgent treatment decisions [1, 2]. In emergency departments, head computed tomography (CT) is the frontline imaging modality for identifying these potentially life-threatening conditions [2, 3]. While CT interpretations are detailed in radiology reports, the free-text format can hinder rapid data extraction and limit its utility for research, quality assurance, and clinical decision support. Converting free-text reports into structured formats has thus become a key objective in contemporary radiologic practice [4, 5].

Recent advances in natural language processing have spurred the development of large language models (LLMs), which can interpret free text with remarkable accuracy and nuance [6–8]. Unlike earlier rule-based or classical machine learning approaches, LLMs leverage massive training corpora to learn contextual relationships, enabling them to identify subtle linguistic cues indicative of pathology [9]. However, their performance can vary considerably depending on model architecture, training data, and prompt design, prompting researchers to investigate optimal strategies for extracting clinically relevant information from free-text radiology reports [9–17]. To the best of our knowledge, no study has comprehensively evaluated multiple LLMs across different prompting approaches in structuring radiology reports written in Japanese [14, 18].

In this study, we aimed to compare the diagnostic performance of three proprietary LLMs—Claude, GPT, and Gemini—in structuring free-text radiology reports of head CT scans for intracranial hemorrhage and skull fractures. Specifically, our classification task focuses on determining the presence or absence of these critical pathologies, rather than identifying their location, size, or orientation. We also examined three distinct prompt approaches, given that prompt engineering may influence model accuracy. By conducting a retrospective evaluation of reports from a multicenter Japanese database, we sought to quantify the models’ performance and identify potential sources of misclassification. Although the task may appear straightforward compared to comprehensive structured reporting (which includes positioning, orientation, and anatomical assessment), accurately extracting these key findings is critical for timely patient management and quality assurance [1, 2, 19].

## Methods

### Study design

**Fig. 1:**
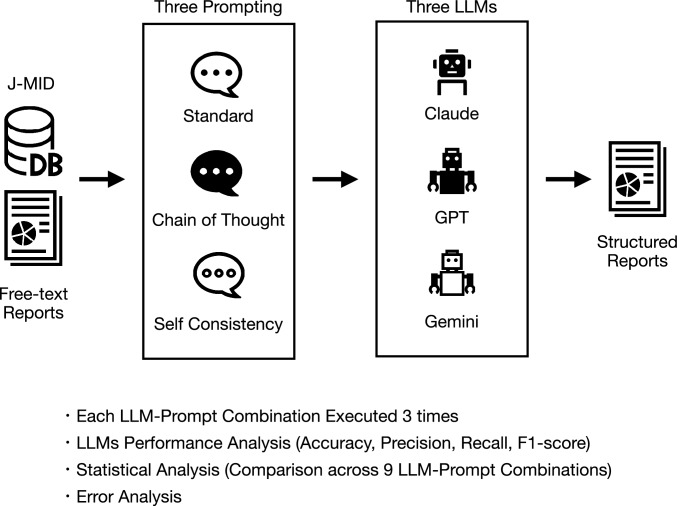
Overview of the study design for evaluating large language model (LLM) performance in structured reporting of head CT findings. Free-text radiology reports were collected from the Japan Medical Imaging Database (J-MID), focusing on emergency department head CT reports from 2018 to 2023. LLM analysis using three different models (Claude 3.5 Sonnet, GPT-4o, and Gemini 1.5 pro) and three prompt types (Standard, Chain of Thought, and Self Consistency), with each combination executed three times for structured reporting of intracranial hemorrhage and skull fracture. LLMs’ performance was evaluated through accuracy metrics, statistical analysis across combinations, and error analysis

In this retrospective study, LLMs were used to structure free-text radiology reports of head CT scans for the presence of intracranial hemorrhage and skull fractures. The reports were structured using three prompt approaches for each of the three LLMs, and their respective diagnostic performances were calculated and statistically compared. An error analysis was then conducted to investigate the underlying causes of any misclassifications. Figure 1 summarizes the study design. The design of this study followed the Standards for Reporting of Diagnostic Accuracy Studies (STARD) guidelines [20].

### Data collection

Free-text radiology reports of head CT scans were consecutively collected from the Japan Medical Imaging Database (J-MID) between 2018 and 2023 [21]. The reports were written in Japanese and were not translated into English. This study focused on head CT scans ordered by the Department of Emergency Medicine, aiming to structure the reports for intracranial hemorrhage and skull fractures. We included all head CT scans ordered by the emergency department without restricting them by clinical indication. When multiple examinations were performed for the same patient, only the initial scan was included in the analysis. Reports were excluded if they met any of the following criteria: (1) absence of imaging findings, (2) unspecified location of hemorrhage or fracture (e.g., cases where hemorrhage or fracture was noted without specifying whether it was intracranial hemorrhage or skull fracture; cervical spine fractures were not considered positive findings in this study), or (3) lack of interpretation regarding "hyperdense areas" (e.g., cases where "hyperdense areas" were noted without specifying whether they represented intracranial hemorrhage), as these omissions precluded determination of intracranial hemorrhage and skull fractures.

### Ground truth

Two board-certified radiologists (H.T. and D.U., each with 10 years of experience) independently reviewed each radiology report to determine the presence or absence of intracranial hemorrhage and skull fracture. In cases of disagreement, the two radiologists discussed until consensus was reached. This expert consensus served as the ground truth.

### Definitions of target conditions

#### Intracranial hemorrhage

For intracranial hemorrhage classification, reports were considered positive if they contained any statement indicating that intracranial hemorrhage was present or possible. Reports were considered negative if they explicitly stated that hemorrhage was absent (e.g., “no hemorrhage,” “hemorrhage not identified”) or did not mention hemorrhage at all.

#### Skull fracture

For skull fractures, reports were considered positive if they contained any statement indicating that a skull fracture was present or possible. Reports were considered negative if they stated the absence of skull fracture (e.g., “no skull fracture,” “skull fracture not identified”) or did not mention skull fracture at all. Bone deformity or defect were not classified as a skull fracture.

#### Large language models and prompts

We used three LLMs (Claude [claude-3.5-sonnet-20240620], GPT [gpt-4o-2024-08-06], and Gemini [gemini-1.5-pro-002]) accessed between October 25 and November 5, 2024 via their application programming interface. Models’ temperatures were set at 0 to limit randomness. Each model analyzed the free-text radiology reports using three distinct prompting strategies: (1) Standard prompting: Direct instructions for classification [22], (2) Chain of Thought prompting: Requests intermediate reasoning steps before the classification [22], (3) Self Consistency prompting: Generation of multiple reasoning paths and selection of the most frequently reached conclusion [23]. The prompts were originally written in Japanese, and the exact Japanese text is provided in Supplementary Methods 1. This resulted in a total of nine test combinations (three LLMs × three prompt types). Each combination was applied to the same set of radiology reports three times independently to account for potential variability in LLM outputs. In each run, the LLM indicated the presence or absence of intracranial hemorrhage and skull fracture, and these classifications were compared to the ground truth.

### Statistical analysis

For each of the nine LLM–prompt combinations, diagnostic performance metrics—including accuracy, precision, recall, and F1-score—were calculated separately for intracranial hemorrhage and skull fracture. Each metric was computed across the three repeated runs and then averaged. Ninety-five percent confidence intervals were calculated for each metric by a bootstrap method (bootstrap sample size = 1000).

Pairwise comparisons across the nine LLM–prompt combinations were performed using McNemar’s test to identify specific differences. Because both intracranial hemorrhage and skull fracture were evaluated (36 pairwise comparisons each, totaling 72 comparisons), a Bonferroni correction was applied (*p* = 0.05/72). Thus, *p*-values < 0.05/72 were considered statistically significant. In addition, a stratified analysis by institution was performed. Detailed methods for this inter-institution comparison are provided in Supplementary Methods 2. Comparison metrics across three independent runs were performed to assess reproducibility. Detailed methods for this comparison are described in Supplementary Methods 3. All statistical analyses were performed using Python version 3.8.2 (Python Software Foundation, https://python.org) with the statsmodels package version 0.13.2 (https://statsmodels.org).

### Error analysis

For each LLM–prompt combination, cases in which the model outputs disagreed with the ground truth were systematically reviewed. All misclassified cases were grouped into categories to provide qualitative insights into recurring error patterns. We further divided misclassified cases into false-positive and false-negative, thereby clarifying whether specific expressions led to over- or under-detection. An institution-specific error pattern analysis was also performed.

## Results

### Participant flow and characteristics

**Fig. 2:**
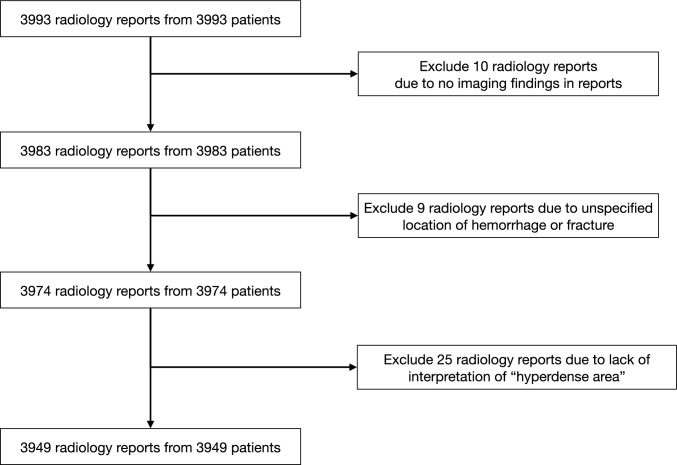
Flow diagram of head CT radiology report selection. From an initial dataset of 3,993 radiology reports, 44 reports were excluded based on three criteria: 10 reports had no descriptions, 9 reports lacked hemorrhage or fracture location information, and 25 reports had no interpretation of high attenuation areas. The final analysis included 3,949 radiology reports from 3,949 unique patients

A total of 3,993 head CT reports met the initial inclusion criteria (Fig. 2). Of these, 44 were excluded, resulting in 3,949 reports for analysis. The demographic characteristics (Table 1) show a mean (± SD) age of 59 ± 25 years, with 56.2% (n = 2,220) of patients being male. Across all institutions, 856 patients (21.6%) had a reference-standard diagnosis of intracranial hemorrhage, and 264 patients (6.6%) had skull fractures.

**Table 1:** Demographics of the source dataset

	Institution A	Institution B	Institution C	Total
Cases	1762	727	1460	3949
Male	948	420	852	2220
Female	814	307	608	1729
Age (mean ± SD)	57 ± 24	52 ± 26	64 ± 25	59 ± 25
Intracranial hemorrhage	103 (5.8%)	336 (46.2%)	417 (28.5%)	856 (21.6%)
Skull fracture	107 (6.0%)	106 (14.5%)	51 (3.5%)	264 (6.6%)

SD standard deviation

### Diagnostic accuracy of each LLM–prompt combination

Diagnostic performance metrics (accuracy, precision, recall, and F1-score) for hemorrhage and skull fracture, along with 95% confidence intervals, are detailed in Table 2. Overall, all nine LLM–prompt configurations demonstrated high accuracy for both intracranial hemorrhage and skull fractures, with only minor variations observed among the different prompt types. Claude 3.5 Sonnet emerged as the top performer, maintaining exceptional consistency across all metrics. In both intracranial hemorrhage and skull fracture, Claude 3.5 Sonnet achieved accuracy, precision, recall, and F1-score ranging from 0.95 to 0.99. GPT-4o demonstrated strong but slightly lower performance compared to Claude 3.5 Sonnet. Gemini 1.5 pro, while still performing well, showed somewhat lower metrics compared to the other two models.

**Table 2:** Diagnostic performance of three large language models

LLM	Label	Prompt	Accuracy	Precision	Recall	F1-score
Claude 3.5 Sonnet	Intracranial Hemorrhage	Standard	0.99 (0.98–0.99)	0.99 (0.98–0.99)	0.96 (0.96–0.97)	0.98 (0.97–0.98)
		Chain of Thought	0.98 (0.98–0.99)	0.98 (0.98–0.99)	0.95 (0.95–0.96)	0.97 (0.96–0.97)
		Self Consistency	0.98 (0.98–0.99)	0.99 (0.98–0.99)	0.95 (0.94–0.96)	0.97 (0.96–0.97)
	Skull Fracture	Standard	0.99 (0.99–0.99)	0.99 (0.98–0.99)	0.96 (0.95–0.98)	0.98 (0.97–0.98)
		Chain of Thought	0.99 (0.99–0.99)	0.99 (0.98–0.99)	0.96 (0.95–0.98)	0.98 (0.97–0.98)
		Self Consistency	0.99 (0.99–0.99)	0.98 (0.97–0.99)	0.96 (0.95–0.98)	0.97 (0.97–0.98)
GPT-4o	Intracranial Hemorrhage	Standard	0.97 (0.97–0.97)	0.99 (0.99–1.0)	0.88 (0.86–0.89)	0.93 (0.92–0.94)
		Chain of Thought	0.97 (0.97–0.97)	0.99 (0.99–0.99)	0.89 (0.87–0.90)	0.94 (0.93–0.94)
		Self Consistency	0.97 (0.97–0.97)	0.99 (0.99–0.99)	0.89 (0.88–0.90)	0.94 (0.93–0.94)
	Skull Fracture	Standard	0.99 (0.99–0.99)	0.97 (0.96–0.98)	0.95 (0.94–0.97)	0.96 (0.95–0.97)
		Chain of Thought	0.99 (0.99–0.99)	0.97 (0.96–0.98)	0.96 (0.95–0.98)	0.97 (0.96–0.98)
		Self Consistency	0.99 (0.99–0.99)	0.97 (0.96–0.98)	0.95 (0.94–0.97)	0.96 (0.95–0.97)
Gemini 1.5 pro	Intracranial Hemorrhage	Standard	0.97 (0.96–0.97)	0.92 (0.90–0.92)	0.95 (0.94–0.96)	0.93 (0.93–0.94)
		Chain of Thought	0.97 (0.97–0.97)	0.93 (0.92–0.94)	0.94 (0.93–0.95)	0.94 (0.93–0.94)
		Self Consistency	0.97 (0.96–0.97)	0.93 (0.92–0.94)	0.94 (0.93–0.94)	0.93 (0.92–0.94)
	Skull Fracture	Standard	0.98 (0.98–0.99)	0.87 (0.85–0.89)	0.97 (0.96–0.98)	0.92 (0.91–0.93)
		Chain of Thought	0.99 (0.98–0.99)	0.89 (0.86–0.90)	0.98 (0.97–0.99)	0.93 (0.92–0.94)
		Self Consistency	0.98 (0.98–0.98)	0.86 (0.83–0.88)	0.98 (0.97–0.99)	0.91 (0.90–0.93)

LLM large language model

### Pairwise comparisons

Table 3 summarizes the results of the pairwise comparisons for intracranial hemorrhage classification. In many comparisons, Claude demonstrated significantly higher accuracy than both GPT and Gemini (*p* < 0.0001), while no statistically significant difference was observed between GPT and Gemini. In contrast, a different trend emerged for skull fracture classification (Table 4): there was no significant difference in accuracy between Claude and GPT, but Gemini exhibited significantly lower accuracy than both Claude and GPT (*p* < 0.0001). For both intracranial hemorrhage and skull fracture classification, Claude and GPT maintained relatively consistent accuracy across different prompting methods, whereas Gemini achieved significantly higher accuracy with Chain of Thought prompting than with either Standard or Self Consistency prompting. Detailed results of the inter-institution comparison are provided in Supplementary Results 1 and Supplementary Table 1. Results of the comparisons across three independent runs are provided in Supplementary Results 2 and Supplementary Table 2.

**Table 3:** Comparison across nine large language model-prompt combinations for structured reporting of intracranial hemorrhage

Model A	Prompt A	Model B	Prompt B	P value	Best Performing
Claude	Chain of Thought	Claude	Standard	0.01	No significant difference
Claude	Chain of Thought	Claude	Self Consistency	1.0	No significant difference
Claude	Chain of Thought	GPT	Chain of Thought	< 0.0001	Claude Chain of Thought
Claude	Chain of Thought	GPT	Standard	< 0.0001	Claude Chain of Thought
Claude	Chain of Thought	GPT	Self Consistency	< 0.0001	Claude Chain of Thought
Claude	Chain of Thought	Gemini	Chain of Thought	0.002	No significant difference
Claude	Chain of Thought	Gemini	Standard	< 0.0001	Claude Chain of Thought
Claude	Chain of Thought	Gemini	Self Consistency	< 0.0001	Claude Chain of Thought
Claude	Standard	Claude	Self Consistency	0.007	No significant difference
Claude	Standard	GPT	Chain of Thought	< 0.0001	Claude Standard
Claude	Standard	GPT	Standard	< 0.0001	Claude Standard
Claude	Standard	GPT	Self Consistency	< 0.0001	Claude Standard
Claude	Standard	Gemini	Chain of Thought	< 0.0001	Claude Standard
Claude	Standard	Gemini	Standard	< 0.0001	Claude Standard
Claude	Standard	Gemini	Self Consistency	< 0.0001	Claude Standard
Claude	Self Consistency	GPT	Chain of Thought	< 0.0001	Claude Self Consistency
Claude	Self Consistency	GPT	Standard	< 0.0001	Claude Self Consistency
Claude	Self Consistency	GPT	Self Consistency	< 0.0001	Claude Self Consistency
Claude	Self Consistency	Gemini	Chain of Thought	0.002	No significant difference
Claude	Self Consistency	Gemini	Standard	< 0.0001	Claude Self Consistency
Claude	Self Consistency	Gemini	Self Consistency	< 0.0001	Claude Self Consistency
GPT	Chain of Thought	GPT	Standard	0.11	No significant difference
GPT	Chain of Thought	GPT	Self Consistency	0.86	No significant difference
GPT	Chain of Thought	Gemini	Chain of Thought	0.01	No significant difference
GPT	Chain of Thought	Gemini	Standard	0.01	No significant difference
GPT	Chain of Thought	Gemini	Self Consistency	0.03	No significant difference
GPT	Standard	GPT	Self Consistency	0.07	No significant difference
GPT	Standard	Gemini	Chain of Thought	0.001	No significant difference
GPT	Standard	Gemini	Standard	0.05	No significant difference
GPT	Standard	Gemini	Self Consistency	0.15	No significant difference
GPT	Self Consistency	Gemini	Chain of Thought	0.02	No significant difference
GPT	Self Consistency	Gemini	Standard	0.005	No significant difference
GPT	Self Consistency	Gemini	Self Consistency	0.02	No significant difference
Gemini	Chain of Thought	Gemini	Standard	< 0.0001	Gemini Chain of Thought
Gemini	Chain of Thought	Gemini	Self Consistency	< 0.0001	Gemini Chain of Thought
Gemini	Standard	Gemini	Self Consistency	0.47	No significant difference

**Table 4:** Comparison across nine large language model-prompt combinations for structured reporting of skull fracture

Model A	Prompt A	Model B	Prompt B	P value	Best Performing
Claude	Chain of Thought	Claude	Standard	1.0	No significant difference
Claude	Chain of Thought	Claude	Self Consistency	1.0	No significant difference
Claude	Chain of Thought	GPT	Chain of Thought	0.38	No significant difference
Claude	Chain of Thought	GPT	Standard	0.17	No significant difference
Claude	Chain of Thought	GPT	Self Consistency	0.05	No significant difference
Claude	Chain of Thought	Gemini	Chain of Thought	0.002	No significant difference
Claude	Chain of Thought	Gemini	Standard	< 0.0001	Claude Chain of Thought
Claude	Chain of Thought	Gemini	Self Consistency	< 0.0001	Claude Chain of Thought
Claude	Standard	Claude	Self Consistency	1.0	No significant difference
Claude	Standard	GPT	Chain of Thought	0.38	No significant difference
Claude	Standard	GPT	Standard	0.17	No significant difference
Claude	Standard	GPT	Self Consistency	0.05	No significant difference
Claude	Standard	Gemini	Chain of Thought	0.002	No significant difference
Claude	Standard	Gemini	Standard	< 0.0001	Claude Standard
Claude	Standard	Gemini	Self Consistency	< 0.0001	Claude Standard
Claude	Self Consistency	GPT	Chain of Thought	0.58	No significant difference
Claude	Self Consistency	GPT	Standard	0.30	No significant difference
Claude	Self Consistency	GPT	Self Consistency	0.11	No significant difference
Claude	Self Consistency	Gemini	Chain of Thought	0.005	No significant difference
Claude	Self Consistency	Gemini	Standard	< 0.0001	Claude Self Consistency
Claude	Self Consistency	Gemini	Self Consistency	< 0.0001	Claude Self Consistency
GPT	Chain of Thought	GPT	Standard	0.68	No significant difference
GPT	Chain of Thought	GPT	Self Consistency	0.28	No significant difference
GPT	Chain of Thought	Gemini	Chain of Thought	0.02	No significant difference
GPT	Chain of Thought	Gemini	Standard	< 0.0001	GPT Chain of Thought
GPT	Chain of Thought	Gemini	Self Consistency	< 0.0001	GPT Chain of Thought
GPT	Standard	GPT	Self Consistency	0.68	No significant difference
GPT	Standard	Gemini	Chain of Thought	0.06	No significant difference
GPT	Standard	Gemini	Standard	< 0.0001	GPT Standard
GPT	Standard	Gemini	Self Consistency	< 0.0001	GPT Standard
GPT	Self Consistency	Gemini	Chain of Thought	0.16	No significant difference
GPT	Self Consistency	Gemini	Standard	< 0.0001	GPT Self Consistency
GPT	Self Consistency	Gemini	Self Consistency	< 0.0001	GPT Self Consistency
Gemini	Chain of Thought	Gemini	Standard	< 0.0001	Gemini Chain of Thought
Gemini	Chain of Thought	Gemini	Self Consistency	< 0.0001	Gemini Chain of Thought
Gemini	Standard	Gemini	Self Consistency	0.80	No significant difference

### Error analysis

Table 5 presents the anticipated causes of misclassification for both intracranial hemorrhage and skull fracture, with errors further categorized as false negatives or false positives. Ambiguous phrases (e.g., “possible”) were associated with errors in both classification tasks. In the case of intracranial hemorrhage, descriptors such as “unchanged hemorrhage”, or “hemorrhage reduction” were associated with false-negative errors, whereas expressions such as “hypodense areas,” “cannot be identified,” or “extracranial hemorrhage” were linked to false-positive errors. Although “incorrect interpretation despite clear description” could, in theory, lead to either error type, in intracranial hemorrhage all such instances were false negatives. Conversely, for skull fracture, while expressions such as “unchanged fracture” tended to yield false negatives, references to “post-surgical changes,” “cervical vertebra fractures,” or “hemorrhage” resulted in false-positive errors. Notably, skull fracture classification showed a higher prevalence of false-positive errors relative to false negatives.

**Table 5:** Error analyses of large language models’ structured reporting

Label	Type	Expected cause of incorrect interpretation	Count (case)
Hemorrhage	False negative	Incorrect interpretation despite clear hemorrhage description	63
	False negative	Contains descriptions of unchanged hemorrhage	58
	False negative	Contains descriptions of hemorrhage reduction/improvement/decreased density	8
	False negative	Contains expressions such as possible/cannot be ruled out/suspected	2
	False positive	Contains expressions such as cannot be identified	69
	False positive	Contains descriptions of edema/swelling/hypodense area/cerebral infarction	39
	False positive	Contains descriptions of extracranial hemorrhage	35
	False positive	Contains descriptions of hemorrhage becoming unclear	30
	False positive	Contains descriptions of intracranial mass	15
	False positive	Contains descriptions of post-hemorrhagic scarring	4
	False positive	Contains descriptions of pseudo-subarachnoid hemorrhage	3
	False positive	Contains descriptions of post-hemorrhage removal status	2
	False positive	Contains descriptions of thrombosis/hyperdense sign	2
	False positive	Contains descriptions of fractures	2
	False positive	Contains descriptions of contrast extravasation	1
	False positive	Contains descriptions of laminar necrosis	1
Fracture	False negative	Incorrect interpretation despite clear fracture description	9
	False negative	Contains descriptions of unchanged fracture	5
	False negative	Contains descriptions of old fracture	3
	False negative	Contains descriptions of post-fracture surgery status	3
	False positive	Contains descriptions of post-craniotomy changes	21
	False positive	Contains expressions such as cannot be identified	9
	False positive	Contains descriptions of cervical vertebra fracture	8
	False positive	Contains descriptions of contusion/hemorrhage	8
	False positive	Contains descriptions of bone defect/destruction	8
	False positive	Contains descriptions of bone mass	4
	False positive	Contains descriptions of nasal septum deviation/nasal bone deformity	3
	False positive	Incorrect interpretation despite clear fracture description	1
	False positive	Contains descriptions of basilar invagination	1
	False positive	Contains descriptions of soft tissue density in paranasal sinuses/temporal bone	1
	False positive	Contains descriptions of unclear fracture	1

Expected cause of incorrect interpretation may contain duplicates

## Discussion

In this retrospective study, three LLMs—Claude, GPT, and Gemini—were evaluated using three distinct prompting strategies for structuring free-text head CT radiology reports in terms of intracranial hemorrhage and skull fractures. The results demonstrated consistently high diagnostic accuracy for each LLM–prompt combination, with slight but noteworthy performance variations. These findings underscore the potential of LLMs for automated classification of key radiological findings, while also highlighting the importance of proper prompting strategies and the need to understand the causes of misclassification.

A central outcome of this study is that all LLMs exhibited strong performance in classifying both intracranial hemorrhage and skull fractures. This underscores the feasibility of leveraging these models for high-level text analysis in radiology. Although the precise architectures and training datasets of the proprietary LLMs were not fully disclosed, their performance validates the potential utility of large-scale language modeling in medical text interpretation, particularly for standardizing free-text reports [11, 24]. The relatively high accuracies observed suggest that such systems could serve as rapid data extraction, quality assurance, and clinical decision support.

Despite this generally strong performance, statistically significant differences emerged across the models. For intracranial hemorrhage classification, Claude demonstrated significantly higher accuracy than GPT and Gemini; in skull fracture classification, Claude also significantly outperformed Gemini. These findings align with earlier benchmark evaluations including reasoning, reading comprehension, and question answering [25]. Furthermore, in structuring free-text radiology reports, some studies showed that Claude-based models outperformed GPT and Gemini in various tasks [26–28]. Although LLMs’ performance can vary depending on their version and specific tasks, these findings suggest that Claude may be useful specifically for structuring free-text radiology reports.

A noteworthy pattern across all LLMs was their sensitivity to the prompting strategy. In this study, Chain of Thought prompting [22], which guides the model through a sequence of intermediate reasoning steps, generally improved performance compared with the Standard prompt, particularly for Gemini. However, Chain of Thought did not enhance the performance of Claude and GPT compared with the Standard prompt. The performance of Claude and GPT with the Standard prompt was significantly higher than that of Gemini, suggesting that baseline performance may shape the extent to which more advanced prompting strategies can yield improvements. Here, Gemini’s baseline (Standard) performance is lower than that of Claude or GPT, which leaves greater room for improvement when more advanced prompting is applied. Conversely, because Claude and GPT already achieve relatively high accuracy with the Standard prompt, the margin for additional gains is more limited. Furthermore, when the baseline performance is relatively low, even moderate improvements achieved by more advanced prompting strategies can result in statistically significant differences, highlighting how baseline performance can magnify the measured impact of each strategy. These findings underscore the importance of considering baseline performance when evaluating the effectiveness of different prompting strategies.

The error analysis revealed common themes responsible for misclassifications. One key source of error was ambiguous language, such as phrases indicating that a hemorrhage or fracture “cannot be excluded.” Although such cautious language is common in clinical reporting—particularly in emergent situations where definitive exclusions may be difficult—it poses inherent challenges for automated classification, which often relies on more binary determinations (i.e., presence vs. absence) [11]. Additionally, references to postoperative changes or mention of other fractures (e.g., cervical vertebra fractures) also contributed to misclassification by diverting the model’s focus. These findings suggest that refining LLMs to better handle conditional or uncertain statements, as well as clarifying relevant versus irrelevant anatomical sites, can help reduce errors.

This study has limitations. Despite sampling radiology reports from multiple institutions, all reports were in Japanese, limiting the immediate generalizability of these findings to other languages or regions. Moreover, retrospective designs can introduce selection biases, particularly regarding how clinical reports are formulated and how certain pathologies are emphasized in emergency settings [29].

In conclusion, LLMs demonstrate strong potential in structuring free-text head CT radiology reports for key findings like intracranial hemorrhage and skull fractures. Although the choice of LLM and prompting strategy can influence accuracy, overall performance remains promising. Future work should address identified sources of misclassification through improved prompt engineering and multilingual validation. A prospective, real-world evaluation of these systems could further establish their utility and foster safer, more efficient radiological assessments.

## Supplementary Information

Below is the link to the electronic supplementary material.

Supplementary file 1 (PDF 866 KB)
