# Supplementary material for: Comparative performance of large language models in structuring head CT radiology reports: multi-institutional validation study in Japan
Source: Jpn J Radiol. 2025 May 14;43(9):1445–55. doi: 10.1007/s11604-025-01799-1 (PMC12396994; doi:10.1007/s11604-025-01799-1)
Supplement: Supplementary file 1 — (PDF 866 KB) [file 11604_2025_1799_MOESM1_ESM.pdf]

Comparative Performance of Large Language Models in Structuring Head CT Radiology Reports: Multi-institutional Validation Study in Japan

Table of Contents:

Section S1: Supplementary Methods
Supplementary Methods 1: Three prompts used in this study
Supplementary Methods 2: Detailed methods for inter-institution comparison
Supplementary Methods 3: Detailed methods for comparison across three independent runs

Section S2: Supplementary Results
Supplementary Results 1: Comparison diagnostic performance of large language models among three institutions
Supplementary Results 2: Pairwise statistical comparisons of repeated runs
Supplementary Results 3: Error analyses of large language models' structured reporting by institutions

Section S3: Supplementary Tables
Supplementary Table 1: Comparison diagnostic performance of large language models among three institutions
Supplementary Table 2: Pairwise statistical comparisons of repeated runs
Supplementary Table 3: Error analyses of large language models' structured reporting by institutions

Section S1: Supplementary Methods

Supplementary Methods 1: Three prompts used in this study
We provide three prompting (Standard, Chain of Thought, Self Consistency) used in this study.

Standard prompting
In Japanese
"今から外傷後に撮影された頭部 CT の放射線科医師のレポートを提示します。
あなたは、研究者としてレポートのデータから外傷による合併症の有無を構造化するサポートをしてください。
放射線科レポートに現時点で頭蓋内出血（ich）または骨折（fracture）の所見があるかどうかを判断し、'ich: True/False, fracture: True/False'の形式のみで回答してください。
また、形式として True/False 以外の文章やコメントは一切必要ありません。区切りはカンマであり、改行を用いるべきではありません。
頭蓋内出血（ich）にはくも膜下出血(SAH)、脳室内出血、脳出血、硬膜下血腫、硬膜外血腫などが含まれます。外耳道や副鼻腔の血腫などは含まれません。
骨折に関しては頭蓋骨や顔面骨の骨折を判定してください。頚椎の骨折は含まれません。
過去に出血があっても現在見られない場合は False として下さい。"

In English
"Below is a radiology report from a head CT scan taken after trauma.
As a researcher, please assist in structuring the data from the report to determine the presence or absence of trauma-related complications. Please decide whether there are current findings of intracranial hemorrhage (ich) or fracture (fracture) and respond strictly in the format 'ich: True/False, fracture: True/False.'
No additional text or commentary beyond 'True/False' is required. Use commas as separators and do not include any line breaks.
For intracranial hemorrhage (ich), include subarachnoid hemorrhage (SAH), intraventricular hemorrhage, cerebral hemorrhage, subdural hematoma, and epidural hematoma, but exclude hematomas in the external auditory canal or paranasal sinuses.
For fractures, determine whether there are fractures in the skull or facial bones. Do not consider cervical spine fractures.
If there was bleeding in the past that is no longer present, please answer False."

Chain of Thought prompting
In Japanese
"今から外傷後に撮影された頭部 CT の放射線科医師のレポートを提示します。
以下の手順で考えてください:
1. レポートの内容を注意深く読む
2. 頭蓋内出血の所見があるか確認する
3. 骨折の所見があるか確認する
4. 結果を'ich: True/False, fracture: True/False'の形式でまとめる
また、形式として True/False 以外の文章やコメントは一切必要ありません。区切りはカンマであり、改行を用いるべきではありません。
頭蓋内出血（ich）にはくも膜下出血(SAH)、脳室内出血、脳出血、硬膜下血腫、硬膜外血腫などが含まれます。外耳道や副鼻腔の血腫などは含まれません。
骨折に関しては頭蓋骨や顔面骨の骨折を判定してください。頚椎の骨折は含まれません。
過去に出血があっても現在見られない場合は False として下さい。"

In English
"Below is a radiology report from a head CT scan taken after trauma.
Please follow these steps:
1. Carefully read the report.

2. Determine whether there are findings of intracranial hemorrhage.
3. Determine whether there are findings of fracture.
4. Summarize the results in the format ‘ich: True/False, fracture: True/False.’
No additional text or commentary beyond ‘True/False’ is necessary. Use commas as separators and do not include any line breaks.
For intracranial hemorrhage (ich), include subarachnoid hemorrhage (SAH), intraventricular hemorrhage, cerebral hemorrhage, subdural hematoma, and epidural hematoma, but exclude hematomas in the external auditory canal or paranasal sinuses.
For fractures, consider fractures in the skull or facial bones only. Do not include cervical spine fractures.
If there was bleeding in the past that is no longer present, please answer False.”

Self Consistency prompting
In Japanese
“今から外傷後に撮影された頭部 CT の放射線科医師のレポートを提示します。
以下の放射線科レポートを 5 回分析し、それぞれの分析で頭蓋内出血（ich）と骨折（fracture）の有無を判断してください。
5 回の分析結果をまとめて、最も多く出現した結果を'ich: True/False, fracture: True/False'の形式で回答してください。
形式として True/False 以外の文章やコメントは一切必要ありません。区切りはカンマであり、改行を用いるべきではありません。
頭蓋内出血（ich）にはくも膜下出血(SAH)、脳室内出血、脳出血、硬膜下血腫、硬膜外血腫などが含まれます。外耳道や副鼻腔の血腫などは含まれません。
骨折に関しては頭蓋骨や顔面骨の骨折を判定してください。頚椎の骨折は含まれません。
過去に出血があっても現在見られない場合は False として下さい。”

In English
“Below is a radiology report from a head CT scan taken after trauma.
Please analyze this report five times, and in each analysis, decide whether there are findings of intracranial hemorrhage (ich) or fracture (fracture). After completing the five analyses, select the most frequently occurring result and provide it in the format ‘ich: True/False, fracture: True/False.’
No additional text or commentary beyond ‘True/False’ is necessary. Use commas as separators and do not include any line breaks.
For intracranial hemorrhage (ich), include subarachnoid hemorrhage (SAH), intraventricular hemorrhage, cerebral hemorrhage, subdural hematoma, and epidural hematoma, but exclude hematomas in the external auditory canal or paranasal sinuses.
For fractures, focus on fractures in the skull or facial bones. Fractures of the cervical spine should not be considered.
If there was bleeding in the past that is no longer present, please answer False.”

Supplementary Methods 2: Detailed methods for inter-institution comparison
In this supplementary analysis, we evaluated whether diagnostic performance differed among Institutions A, B, and C. Specifically, we calculated metrics such as accuracy, precision, recall, and F1 measure for each institution and then compared these metrics on a pairwise basis. Statistical significance was assessed using the two-sided Mann-Whitney U test with a nominal alpha level of 0.05. Given that two labels (intracranial hemorrhage and skull fracture) were evaluated using three LLMs with three types of prompts and four metrics across three institutions, a total of $2\times3\times3\times4\times3=216$ statistical comparisons were performed. We applied a Bonferroni correction and considered p values below 0.05/216 to be statistically significant.

Supplementary Methods 3: Detailed methods for comparison across three independent runs
Each LLM–prompt combination was run three times independently. We compared performance metrics (accuracy, precision, recall, and F1) across these runs using the two-sided Mann–Whitney U test with a nominal alpha level of 0.05. Given that two labels (intracranial hemorrhage and skull fracture) were evaluated using three LLMs with three types of prompts and four metrics across three runs, a total of $2\times3\times3\times4\times3=216$ statistical comparisons were performed. We applied a Bonferroni correction and considered p values below 0.05/216 to be statistically significant.

Section S2: Supplementary Results

Supplementary Results 1: Comparison of diagnostic performance of large language models among three institutions

To assess the consistency of the LLM-based structuring approach across different clinical settings, the dataset was stratified by institution. The performance metrics—accuracy, precision, recall, and F1 measure—or the classification of intracranial hemorrhage and skull fractures were computed separately for Institutions A, B, and C (Supplementary Table 1). The metrics were highly consistent across institutions, with no statistically significant differences ($p > 0.05/216$), which supports the universal applicability of our method.

Supplementary Results 2: Pairwise statistical comparisons of repeated runs

In most cases, performance was highly consistent across the three independent runs, despite the temperature being set to 0. However, some metrics showed minor variations that reached statistical significance. In total, 216 statistical comparisons were performed (reflecting two labels, three LLMs, three prompt types, three pairwise run comparisons, and four metrics). Among these, Claude showed significant differences in only 4 comparisons, while GPT and Gemini had 44 and 51, respectively. These findings indicate that Claude exhibited the least run-to-run variability overall. A full summary is provided in Supplementary Table 2.

Supplementary Results 3: Error analyses of large language models' structured reporting by institutions

When we compared error patterns by institution (Supplementary Table 3), we observed several site-specific trends. Although certain sources of error—such as post-craniotomy changes—were present across all three institutions, others, including unchanged hemorrhage or ambiguous expressions such as 'possible hemorrhage,' varied in frequency. These findings suggest that local reporting styles or differences in case mix may influence the distribution of errors.

Section S3: Supplementary Tables

Supplementary Table 1: Comparison of diagnostic performance of large language models among three institutions

Label	LLM	Prompt	Metric	Institution 1	Institution 2	P value	Results
Intracranial Hemorrhage	Claude	Chain of Thought	Accuracy	Institution A	Institution B	0.05	No significant difference
Intracranial Hemorrhage	Claude	Chain of Thought	Accuracy	Institution A	Institution C	0.05	No significant difference
Intracranial Hemorrhage	Claude	Chain of Thought	Accuracy	Institution B	Institution C	0.05	No significant difference
Intracranial Hemorrhage	Claude	Chain of Thought	F1 measure	Institution A	Institution B	0.05	No significant difference
Intracranial Hemorrhage	Claude	Chain of Thought	F1 measure	Institution A	Institution C	0.05	No significant difference
Intracranial Hemorrhage	Claude	Chain of Thought	F1 measure	Institution B	Institution C	0.05	No significant difference
Intracranial Hemorrhage	Claude	Chain of Thought	Precision	Institution A	Institution B	0.05	No significant difference
Intracranial Hemorrhage	Claude	Chain of Thought	Precision	Institution A	Institution C	0.05	No significant difference
Intracranial Hemorrhage	Claude	Chain of Thought	Precision	Institution B	Institution C	0.05	No significant difference
Intracranial Hemorrhage	Claude	Chain of Thought	Recall	Institution A	Institution B	0.05	No significant difference
Intracranial Hemorrhage	Claude	Chain of Thought	Recall	Institution A	Institution C	0.05	No significant difference
Intracranial Hemorrhage	Claude	Chain of Thought	Recall	Institution B	Institution C	0.05	No significant difference
Intracranial Hemorrhage	Claude	Self Consistency	Accuracy	Institution A	Institution B	0.05	No significant difference
Intracranial Hemorrhage	Claude	Self Consistency	Accuracy	Institution A	Institution C	0.05	No significant difference
Intracranial Hemorrhage	Claude	Self Consistency	Accuracy	Institution B	Institution C	0.05	No significant difference
Intracranial Hemorrhage	Claude	Self Consistency	F1 measure	Institution A	Institution B	0.05	No significant difference
Intracranial Hemorrhage	Claude	Self Consistency	F1 measure	Institution A	Institution C	0.05	No significant difference
Intracranial Hemorrhage	Claude	Self Consistency	F1 measure	Institution B	Institution C	0.05	No significant difference
Intracranial Hemorrhage	Claude	Self Consistency	Precision	Institution A	Institution B	0.05	No significant difference
Intracranial Hemorrhage	Claude	Self Consistency	Precision	Institution A	Institution C	0.05	No significant difference
Intracranial Hemorrhage	Claude	Self Consistency	Precision	Institution B	Institution C	0.05	No significant difference
Intracranial Hemorrhage	Claude	Self Consistency	Recall	Institution A	Institution B	0.05	No significant difference
Intracranial Hemorrhage	Claude	Self Consistency	Recall	Institution A	Institution C	0.05	No significant difference
Intracranial Hemorrhage	Claude	Self Consistency	Recall	Institution B	Institution C	0.05	No significant difference
Intracranial Hemorrhage	Claude	Standard	Accuracy	Institution A	Institution B	0.07	No significant difference
Intracranial Hemorrhage	Claude	Standard	Accuracy	Institution A	Institution C	0.06	No significant difference
Intracranial Hemorrhage	Claude	Standard	Accuracy	Institution B	Institution C	0.06	No significant difference
Intracranial Hemorrhage	Claude	Standard	F1 measure	Institution A	Institution B	0.07	No significant difference
Intracranial Hemorrhage	Claude	Standard	F1 measure	Institution A	Institution C	0.06	No significant difference
Intracranial Hemorrhage	Claude	Standard	F1 measure	Institution B	Institution C	0.06	No significant difference
Intracranial Hemorrhage	Claude	Standard	Precision	Institution A	Institution B	0.07	No significant difference
Intracranial Hemorrhage	Claude	Standard	Precision	Institution A	Institution C	0.64	No significant difference
Intracranial Hemorrhage	Claude	Standard	Precision	Institution B	Institution C	0.06	No significant difference
Intracranial Hemorrhage	Claude	Standard	Recall	Institution A	Institution B	0.06	No significant difference
Intracranial Hemorrhage	Claude	Standard	Recall	Institution A	Institution C	0.05	No significant difference

Intracranial Hemorrhage	Claude	Standard	Recall	Institution B	Institution C	0.06	No significant difference
Intracranial Hemorrhage	GPT	Chain of Thought	Accuracy	Institution A	Institution B	0.1	No significant difference
Intracranial Hemorrhage	GPT	Chain of Thought	Accuracy	Institution A	Institution C	0.1	No significant difference
Intracranial Hemorrhage	GPT	Chain of Thought	Accuracy	Institution B	Institution C	0.1	No significant difference
Intracranial Hemorrhage	GPT	Chain of Thought	F1 measure	Institution A	Institution B	0.1	No significant difference
Intracranial Hemorrhage	GPT	Chain of Thought	F1 measure	Institution A	Institution C	0.1	No significant difference
Intracranial Hemorrhage	GPT	Chain of Thought	F1 measure	Institution B	Institution C	0.1	No significant difference
Intracranial Hemorrhage	GPT	Chain of Thought	Precision	Institution A	Institution B	1	No significant difference
Intracranial Hemorrhage	GPT	Chain of Thought	Precision	Institution A	Institution C	0.5	No significant difference
Intracranial Hemorrhage	GPT	Chain of Thought	Precision	Institution B	Institution C	0.2	No significant difference
Intracranial Hemorrhage	GPT	Chain of Thought	Recall	Institution A	Institution B	0.1	No significant difference
Intracranial Hemorrhage	GPT	Chain of Thought	Recall	Institution A	Institution C	0.1	No significant difference
Intracranial Hemorrhage	GPT	Chain of Thought	Recall	Institution B	Institution C	0.1	No significant difference
Intracranial Hemorrhage	GPT	Self Consistency	Accuracy	Institution A	Institution B	0.08	No significant difference
Intracranial Hemorrhage	GPT	Self Consistency	Accuracy	Institution A	Institution C	0.07	No significant difference
Intracranial Hemorrhage	GPT	Self Consistency	Accuracy	Institution B	Institution C	0.08	No significant difference
Intracranial Hemorrhage	GPT	Self Consistency	F1 measure	Institution A	Institution B	0.08	No significant difference
Intracranial Hemorrhage	GPT	Self Consistency	F1 measure	Institution A	Institution C	0.07	No significant difference
Intracranial Hemorrhage	GPT	Self Consistency	F1 measure	Institution B	Institution C	0.08	No significant difference
Intracranial Hemorrhage	GPT	Self Consistency	Precision	Institution A	Institution B	0.06	No significant difference
Intracranial Hemorrhage	GPT	Self Consistency	Precision	Institution A	Institution C	0.06	No significant difference
Intracranial Hemorrhage	GPT	Self Consistency	Precision	Institution B	Institution C	1	No significant difference
Intracranial Hemorrhage	GPT	Self Consistency	Recall	Institution A	Institution B	0.08	No significant difference
Intracranial Hemorrhage	GPT	Self Consistency	Recall	Institution A	Institution C	0.07	No significant difference
Intracranial Hemorrhage	GPT	Self Consistency	Recall	Institution B	Institution C	0.08	No significant difference
Intracranial Hemorrhage	GPT	Standard	Accuracy	Institution A	Institution B	0.1	No significant difference
Intracranial Hemorrhage	GPT	Standard	Accuracy	Institution A	Institution C	0.08	No significant difference
Intracranial Hemorrhage	GPT	Standard	Accuracy	Institution B	Institution C	0.08	No significant difference
Intracranial Hemorrhage	GPT	Standard	F1 measure	Institution A	Institution B	0.1	No significant difference
Intracranial Hemorrhage	GPT	Standard	F1 measure	Institution A	Institution C	0.08	No significant difference
Intracranial Hemorrhage	GPT	Standard	F1 measure	Institution B	Institution C	0.08	No significant difference
Intracranial Hemorrhage	GPT	Standard	Precision	Institution A	Institution B	0.2	No significant difference
Intracranial Hemorrhage	GPT	Standard	Precision	Institution A	Institution C	0.2	No significant difference
Intracranial Hemorrhage	GPT	Standard	Precision	Institution B	Institution C	1	No significant difference
Intracranial Hemorrhage	GPT	Standard	Recall	Institution A	Institution B	0.08	No significant difference
Intracranial Hemorrhage	GPT	Standard	Recall	Institution A	Institution C	0.07	No significant difference
Intracranial Hemorrhage	GPT	Standard	Recall	Institution B	Institution C	0.08	No significant difference
Intracranial Hemorrhage	Gemini	Chain of Thought	Accuracy	Institution A	Institution B	0.37	No significant difference
Intracranial Hemorrhage	Gemini	Chain of Thought	Accuracy	Institution A	Institution C	0.66	No significant difference
Intracranial Hemorrhage	Gemini	Chain of Thought	Accuracy	Institution B	Institution C	0.08	No significant difference

Intracranial Hemorrhage	Gemini	Chain of Thought	F1 measure	Institution A	Institution B	0.07	No significant difference
Intracranial Hemorrhage	Gemini	Chain of Thought	F1 measure	Institution A	Institution C	0.08	No significant difference
Intracranial Hemorrhage	Gemini	Chain of Thought	F1 measure	Institution B	Institution C	0.66	No significant difference
Intracranial Hemorrhage	Gemini	Chain of Thought	Precision	Institution A	Institution B	0.07	No significant difference
Intracranial Hemorrhage	Gemini	Chain of Thought	Precision	Institution A	Institution C	0.08	No significant difference
Intracranial Hemorrhage	Gemini	Chain of Thought	Precision	Institution B	Institution C	0.38	No significant difference
Intracranial Hemorrhage	Gemini	Chain of Thought	Recall	Institution A	Institution B	0.07	No significant difference
Intracranial Hemorrhage	Gemini	Chain of Thought	Recall	Institution A	Institution C	0.65	No significant difference
Intracranial Hemorrhage	Gemini	Chain of Thought	Recall	Institution B	Institution C	0.07	No significant difference
Intracranial Hemorrhage	Gemini	Self Consistency	Accuracy	Institution A	Institution B	0.1	No significant difference
Intracranial Hemorrhage	Gemini	Self Consistency	Accuracy	Institution A	Institution C	0.4	No significant difference
Intracranial Hemorrhage	Gemini	Self Consistency	Accuracy	Institution B	Institution C	0.1	No significant difference
Intracranial Hemorrhage	Gemini	Self Consistency	F1 measure	Institution A	Institution B	0.1	No significant difference
Intracranial Hemorrhage	Gemini	Self Consistency	F1 measure	Institution A	Institution C	0.1	No significant difference
Intracranial Hemorrhage	Gemini	Self Consistency	F1 measure	Institution B	Institution C	0.7	No significant difference
Intracranial Hemorrhage	Gemini	Self Consistency	Precision	Institution A	Institution B	0.1	No significant difference
Intracranial Hemorrhage	Gemini	Self Consistency	Precision	Institution A	Institution C	0.1	No significant difference
Intracranial Hemorrhage	Gemini	Self Consistency	Precision	Institution B	Institution C	0.1	No significant difference
Intracranial Hemorrhage	Gemini	Self Consistency	Recall	Institution A	Institution B	0.08	No significant difference
Intracranial Hemorrhage	Gemini	Self Consistency	Recall	Institution A	Institution C	0.38	No significant difference
Intracranial Hemorrhage	Gemini	Self Consistency	Recall	Institution B	Institution C	0.1	No significant difference
Intracranial Hemorrhage	Gemini	Standard	Accuracy	Institution A	Institution B	0.4	No significant difference
Intracranial Hemorrhage	Gemini	Standard	Accuracy	Institution A	Institution C	1	No significant difference
Intracranial Hemorrhage	Gemini	Standard	Accuracy	Institution B	Institution C	0.4	No significant difference
Intracranial Hemorrhage	Gemini	Standard	F1 measure	Institution A	Institution B	0.1	No significant difference
Intracranial Hemorrhage	Gemini	Standard	F1 measure	Institution A	Institution C	0.1	No significant difference
Intracranial Hemorrhage	Gemini	Standard	F1 measure	Institution B	Institution C	0.7	No significant difference
Intracranial Hemorrhage	Gemini	Standard	Precision	Institution A	Institution B	0.1	No significant difference
Intracranial Hemorrhage	Gemini	Standard	Precision	Institution A	Institution C	0.1	No significant difference
Intracranial Hemorrhage	Gemini	Standard	Precision	Institution B	Institution C	0.4	No significant difference
Intracranial Hemorrhage	Gemini	Standard	Recall	Institution A	Institution B	0.2	No significant difference
Intracranial Hemorrhage	Gemini	Standard	Recall	Institution A	Institution C	0.7	No significant difference
Intracranial Hemorrhage	Gemini	Standard	Recall	Institution B	Institution C	0.2	No significant difference
Skull Fracture	Claude	Chain of Thought	Accuracy	Institution A	Institution B	0.05	No significant difference
Skull Fracture	Claude	Chain of Thought	Accuracy	Institution A	Institution C	0.05	No significant difference
Skull Fracture	Claude	Chain of Thought	Accuracy	Institution B	Institution C	0.05	No significant difference
Skull Fracture	Claude	Chain of Thought	F1 measure	Institution A	Institution B	0.05	No significant difference
Skull Fracture	Claude	Chain of Thought	F1 measure	Institution A	Institution C	0.05	No significant difference
Skull Fracture	Claude	Chain of Thought	F1 measure	Institution B	Institution C	0.05	No significant difference
Skull Fracture	Claude	Chain of Thought	Precision	Institution A	Institution B	0.05	No significant difference

Skull Fracture	Claude	Chain of Thought	Precision	Institution A	Institution C	0.05	No significant difference
Skull Fracture	Claude	Chain of Thought	Precision	Institution B	Institution C	0.05	No significant difference
Skull Fracture	Claude	Chain of Thought	Recall	Institution A	Institution B	0.05	No significant difference
Skull Fracture	Claude	Chain of Thought	Recall	Institution A	Institution C	0.05	No significant difference
Skull Fracture	Claude	Chain of Thought	Recall	Institution B	Institution C	0.05	No significant difference
Skull Fracture	Claude	Self Consistency	Accuracy	Institution A	Institution B	0.05	No significant difference
Skull Fracture	Claude	Self Consistency	Accuracy	Institution A	Institution C	0.05	No significant difference
Skull Fracture	Claude	Self Consistency	Accuracy	Institution B	Institution C	0.05	No significant difference
Skull Fracture	Claude	Self Consistency	F1 measure	Institution A	Institution B	0.05	No significant difference
Skull Fracture	Claude	Self Consistency	F1 measure	Institution A	Institution C	0.05	No significant difference
Skull Fracture	Claude	Self Consistency	F1 measure	Institution B	Institution C	0.05	No significant difference
Skull Fracture	Claude	Self Consistency	Precision	Institution A	Institution B	0.05	No significant difference
Skull Fracture	Claude	Self Consistency	Precision	Institution A	Institution C	0.05	No significant difference
Skull Fracture	Claude	Self Consistency	Precision	Institution B	Institution C	0.05	No significant difference
Skull Fracture	Claude	Self Consistency	Recall	Institution A	Institution B	0.05	No significant difference
Skull Fracture	Claude	Self Consistency	Recall	Institution A	Institution C	0.05	No significant difference
Skull Fracture	Claude	Self Consistency	Recall	Institution B	Institution C	0.05	No significant difference
Skull Fracture	Claude	Standard	Accuracy	Institution A	Institution B	0.05	No significant difference
Skull Fracture	Claude	Standard	Accuracy	Institution A	Institution C	0.05	No significant difference
Skull Fracture	Claude	Standard	Accuracy	Institution B	Institution C	0.05	No significant difference
Skull Fracture	Claude	Standard	F1 measure	Institution A	Institution B	0.05	No significant difference
Skull Fracture	Claude	Standard	F1 measure	Institution A	Institution C	0.05	No significant difference
Skull Fracture	Claude	Standard	F1 measure	Institution B	Institution C	0.05	No significant difference
Skull Fracture	Claude	Standard	Precision	Institution A	Institution B	0.05	No significant difference
Skull Fracture	Claude	Standard	Precision	Institution A	Institution C	0.05	No significant difference
Skull Fracture	Claude	Standard	Precision	Institution B	Institution C	0.05	No significant difference
Skull Fracture	Claude	Standard	Recall	Institution A	Institution B	0.05	No significant difference
Skull Fracture	Claude	Standard	Recall	Institution A	Institution C	0.05	No significant difference
Skull Fracture	Claude	Standard	Recall	Institution B	Institution C	0.05	No significant difference
Skull Fracture	GPT	Chain of Thought	Accuracy	Institution A	Institution B	0.06	No significant difference
Skull Fracture	GPT	Chain of Thought	Accuracy	Institution A	Institution C	0.06	No significant difference
Skull Fracture	GPT	Chain of Thought	Accuracy	Institution B	Institution C	0.08	No significant difference
Skull Fracture	GPT	Chain of Thought	F1 measure	Institution A	Institution B	0.06	No significant difference
Skull Fracture	GPT	Chain of Thought	F1 measure	Institution A	Institution C	0.06	No significant difference
Skull Fracture	GPT	Chain of Thought	F1 measure	Institution B	Institution C	0.18	No significant difference
Skull Fracture	GPT	Chain of Thought	Precision	Institution A	Institution B	0.06	No significant difference
Skull Fracture	GPT	Chain of Thought	Precision	Institution A	Institution C	0.06	No significant difference
Skull Fracture	GPT	Chain of Thought	Precision	Institution B	Institution C	0.08	No significant difference
Skull Fracture	GPT	Chain of Thought	Recall	Institution A	Institution B	0.06	No significant difference
Skull Fracture	GPT	Chain of Thought	Recall	Institution A	Institution C	0.06	No significant difference

Skull Fracture	GPT	Chain of Thought	Recall	Institution B	Institution C	0.07	No significant difference
Skull Fracture	GPT	Self Consistency	Accuracy	Institution A	Institution B	0.06	No significant difference
Skull Fracture	GPT	Self Consistency	Accuracy	Institution A	Institution C	0.06	No significant difference
Skull Fracture	GPT	Self Consistency	Accuracy	Institution B	Institution C	0.07	No significant difference
Skull Fracture	GPT	Self Consistency	F1 measure	Institution A	Institution B	0.06	No significant difference
Skull Fracture	GPT	Self Consistency	F1 measure	Institution A	Institution C	0.06	No significant difference
Skull Fracture	GPT	Self Consistency	F1 measure	Institution B	Institution C	0.08	No significant difference
Skull Fracture	GPT	Self Consistency	Precision	Institution A	Institution B	0.64	No significant difference
Skull Fracture	GPT	Self Consistency	Precision	Institution A	Institution C	0.64	No significant difference
Skull Fracture	GPT	Self Consistency	Precision	Institution B	Institution C	1	No significant difference
Skull Fracture	GPT	Self Consistency	Recall	Institution A	Institution B	0.06	No significant difference
Skull Fracture	GPT	Self Consistency	Recall	Institution A	Institution C	0.05	No significant difference
Skull Fracture	GPT	Self Consistency	Recall	Institution B	Institution C	0.06	No significant difference
Skull Fracture	GPT	Standard	Accuracy	Institution A	Institution B	0.64	No significant difference
Skull Fracture	GPT	Standard	Accuracy	Institution A	Institution C	0.06	No significant difference
Skull Fracture	GPT	Standard	Accuracy	Institution B	Institution C	0.08	No significant difference
Skull Fracture	GPT	Standard	F1 measure	Institution A	Institution B	0.06	No significant difference
Skull Fracture	GPT	Standard	F1 measure	Institution A	Institution C	0.06	No significant difference
Skull Fracture	GPT	Standard	F1 measure	Institution B	Institution C	0.38	No significant difference
Skull Fracture	GPT	Standard	Precision	Institution A	Institution B	0.06	No significant difference
Skull Fracture	GPT	Standard	Precision	Institution A	Institution C	0.06	No significant difference
Skull Fracture	GPT	Standard	Precision	Institution B	Institution C	0.08	No significant difference
Skull Fracture	GPT	Standard	Recall	Institution A	Institution B	0.06	No significant difference
Skull Fracture	GPT	Standard	Recall	Institution A	Institution C	0.06	No significant difference
Skull Fracture	GPT	Standard	Recall	Institution B	Institution C	0.07	No significant difference
Skull Fracture	Gemini	Chain of Thought	Accuracy	Institution A	Institution B	0.38	No significant difference
Skull Fracture	Gemini	Chain of Thought	Accuracy	Institution A	Institution C	0.38	No significant difference
Skull Fracture	Gemini	Chain of Thought	Accuracy	Institution B	Institution C	0.37	No significant difference
Skull Fracture	Gemini	Chain of Thought	F1 measure	Institution A	Institution B	0.38	No significant difference
Skull Fracture	Gemini	Chain of Thought	F1 measure	Institution A	Institution C	0.38	No significant difference
Skull Fracture	Gemini	Chain of Thought	F1 measure	Institution B	Institution C	0.07	No significant difference
Skull Fracture	Gemini	Chain of Thought	Precision	Institution A	Institution B	0.38	No significant difference
Skull Fracture	Gemini	Chain of Thought	Precision	Institution A	Institution C	0.38	No significant difference
Skull Fracture	Gemini	Chain of Thought	Precision	Institution B	Institution C	0.37	No significant difference
Skull Fracture	Gemini	Chain of Thought	Recall	Institution A	Institution B	0.06	No significant difference
Skull Fracture	Gemini	Chain of Thought	Recall	Institution A	Institution C	0.07	No significant difference
Skull Fracture	Gemini	Chain of Thought	Recall	Institution B	Institution C	0.06	No significant difference
Skull Fracture	Gemini	Self Consistency	Accuracy	Institution A	Institution B	0.07	No significant difference
Skull Fracture	Gemini	Self Consistency	Accuracy	Institution A	Institution C	0.65	No significant difference
Skull Fracture	Gemini	Self Consistency	Accuracy	Institution B	Institution C	0.37	No significant difference

Skull Fracture	Gemini	Self Consistency	F1 measure	Institution A	Institution B	0.37	No significant difference
Skull Fracture	Gemini	Self Consistency	F1 measure	Institution A	Institution C	0.37	No significant difference
Skull Fracture	Gemini	Self Consistency	F1 measure	Institution B	Institution C	0.07	No significant difference
Skull Fracture	Gemini	Self Consistency	Precision	Institution A	Institution B	0.49	No significant difference
Skull Fracture	Gemini	Self Consistency	Precision	Institution A	Institution C	0.37	No significant difference
Skull Fracture	Gemini	Self Consistency	Precision	Institution B	Institution C	0.37	No significant difference
Skull Fracture	Gemini	Self Consistency	Recall	Institution A	Institution B	0.05	No significant difference
Skull Fracture	Gemini	Self Consistency	Recall	Institution A	Institution C	0.06	No significant difference
Skull Fracture	Gemini	Self Consistency	Recall	Institution B	Institution C	0.06	No significant difference
Skull Fracture	Gemini	Standard	Accuracy	Institution A	Institution B	0.37	No significant difference
Skull Fracture	Gemini	Standard	Accuracy	Institution A	Institution C	1	No significant difference
Skull Fracture	Gemini	Standard	Accuracy	Institution B	Institution C	0.38	No significant difference
Skull Fracture	Gemini	Standard	F1 measure	Institution A	Institution B	0.37	No significant difference
Skull Fracture	Gemini	Standard	F1 measure	Institution A	Institution C	0.38	No significant difference
Skull Fracture	Gemini	Standard	F1 measure	Institution B	Institution C	0.38	No significant difference
Skull Fracture	Gemini	Standard	Precision	Institution A	Institution B	0.37	No significant difference
Skull Fracture	Gemini	Standard	Precision	Institution A	Institution C	0.38	No significant difference
Skull Fracture	Gemini	Standard	Precision	Institution B	Institution C	0.38	No significant difference
Skull Fracture	Gemini	Standard	Recall	Institution A	Institution B	0.05	No significant difference
Skull Fracture	Gemini	Standard	Recall	Institution A	Institution C	0.06	No significant difference
Skull Fracture	Gemini	Standard	Recall	Institution B	Institution C	0.06	No significant difference

LLM large language model

Supplementary Table 2: Pairwise statistical comparisons of repeated runs

Label	LLM	Prompt	Metric	Run 1	Run 2	P value	Results
Intracranial Hemorrhage	Claude	Chain of Thought	Accuracy	First Run	Second Run	1	No significant difference
Intracranial Hemorrhage	Claude	Chain of Thought	Accuracy	First Run	Third Run	1	No significant difference
Intracranial Hemorrhage	Claude	Chain of Thought	Accuracy	Second Run	Third Run	1	No significant difference
Intracranial Hemorrhage	Claude	Chain of Thought	F1 measure	First Run	Second Run	1	No significant difference
Intracranial Hemorrhage	Claude	Chain of Thought	F1 measure	First Run	Third Run	1	No significant difference
Intracranial Hemorrhage	Claude	Chain of Thought	F1 measure	Second Run	Third Run	1	No significant difference
Intracranial Hemorrhage	Claude	Chain of Thought	Precision	First Run	Second Run	1	No significant difference
Intracranial Hemorrhage	Claude	Chain of Thought	Precision	First Run	Third Run	1	No significant difference
Intracranial Hemorrhage	Claude	Chain of Thought	Precision	Second Run	Third Run	1	No significant difference
Intracranial Hemorrhage	Claude	Chain of Thought	Recall	First Run	Second Run	1	No significant difference
Intracranial Hemorrhage	Claude	Chain of Thought	Recall	First Run	Third Run	1	No significant difference
Intracranial Hemorrhage	Claude	Chain of Thought	Recall	Second Run	Third Run	1	No significant difference
Intracranial Hemorrhage	Claude	Self Consistency	Accuracy	First Run	Second Run	1	No significant difference
Intracranial Hemorrhage	Claude	Self Consistency	Accuracy	First Run	Third Run	1	No significant difference
Intracranial Hemorrhage	Claude	Self Consistency	Accuracy	Second Run	Third Run	1	No significant difference
Intracranial Hemorrhage	Claude	Self Consistency	F1 measure	First Run	Second Run	1	No significant difference
Intracranial Hemorrhage	Claude	Self Consistency	F1 measure	First Run	Third Run	1	No significant difference
Intracranial Hemorrhage	Claude	Self Consistency	F1 measure	Second Run	Third Run	1	No significant difference
Intracranial Hemorrhage	Claude	Self Consistency	Precision	First Run	Second Run	1	No significant difference
Intracranial Hemorrhage	Claude	Self Consistency	Precision	First Run	Third Run	1	No significant difference
Intracranial Hemorrhage	Claude	Self Consistency	Precision	Second Run	Third Run	1	No significant difference
Intracranial Hemorrhage	Claude	Self Consistency	Recall	First Run	Second Run	1	No significant difference
Intracranial Hemorrhage	Claude	Self Consistency	Recall	First Run	Third Run	1	No significant difference
Intracranial Hemorrhage	Claude	Self Consistency	Recall	Second Run	Third Run	1	No significant difference
Intracranial Hemorrhage	Claude	Standard	Accuracy	First Run	Second Run	0.001	No significant difference
Intracranial Hemorrhage	Claude	Standard	Accuracy	First Run	Third Run	0.001	No significant difference
Intracranial Hemorrhage	Claude	Standard	Accuracy	Second Run	Third Run	1	No significant difference
Intracranial Hemorrhage	Claude	Standard	F1 measure	First Run	Second Run	< 0.0001	Significant difference
Intracranial Hemorrhage	Claude	Standard	F1 measure	First Run	Third Run	< 0.0001	Significant difference
Intracranial Hemorrhage	Claude	Standard	F1 measure	Second Run	Third Run	1	No significant difference
Intracranial Hemorrhage	Claude	Standard	Precision	First Run	Second Run	< 0.0001	Significant difference
Intracranial Hemorrhage	Claude	Standard	Precision	First Run	Third Run	< 0.0001	Significant difference
Intracranial Hemorrhage	Claude	Standard	Precision	Second Run	Third Run	1	No significant difference
Intracranial Hemorrhage	Claude	Standard	Recall	First Run	Second Run	0.01	No significant difference
Intracranial Hemorrhage	Claude	Standard	Recall	First Run	Third Run	0.01	No significant difference
Intracranial Hemorrhage	Claude	Standard	Recall	Second Run	Third Run	1	No significant difference
Intracranial Hemorrhage	GPT	Chain of Thought	Accuracy	First Run	Second Run	0.0001	Significant difference

Intracranial Hemorrhage	GPT	Chain of Thought	Accuracy	First Run	Third Run	< 0.0001	Significant difference
Intracranial Hemorrhage	GPT	Chain of Thought	Accuracy	Second Run	Third Run	0.61	No significant difference
Intracranial Hemorrhage	GPT	Chain of Thought	F1 measure	First Run	Second Run	< 0.0001	Significant difference
Intracranial Hemorrhage	GPT	Chain of Thought	F1 measure	First Run	Third Run	< 0.0001	Significant difference
Intracranial Hemorrhage	GPT	Chain of Thought	F1 measure	Second Run	Third Run	0.04	No significant difference
Intracranial Hemorrhage	GPT	Chain of Thought	Precision	First Run	Second Run	< 0.0001	Significant difference
Intracranial Hemorrhage	GPT	Chain of Thought	Precision	First Run	Third Run	< 0.0001	Significant difference
Intracranial Hemorrhage	GPT	Chain of Thought	Precision	Second Run	Third Run	< 0.0001	Significant difference
Intracranial Hemorrhage	GPT	Chain of Thought	Recall	First Run	Second Run	< 0.0001	Significant difference
Intracranial Hemorrhage	GPT	Chain of Thought	Recall	First Run	Third Run	< 0.0001	Significant difference
Intracranial Hemorrhage	GPT	Chain of Thought	Recall	Second Run	Third Run	0.99	No significant difference
Intracranial Hemorrhage	GPT	Self Consistency	Accuracy	First Run	Second Run	0.0001	Significant difference
Intracranial Hemorrhage	GPT	Self Consistency	Accuracy	First Run	Third Run	0.63	No significant difference
Intracranial Hemorrhage	GPT	Self Consistency	Accuracy	Second Run	Third Run	< 0.0001	Significant difference
Intracranial Hemorrhage	GPT	Self Consistency	F1 measure	First Run	Second Run	< 0.0001	Significant difference
Intracranial Hemorrhage	GPT	Self Consistency	F1 measure	First Run	Third Run	0.79	No significant difference
Intracranial Hemorrhage	GPT	Self Consistency	F1 measure	Second Run	Third Run	< 0.0001	Significant difference
Intracranial Hemorrhage	GPT	Self Consistency	Precision	First Run	Second Run	< 0.0001	Significant difference
Intracranial Hemorrhage	GPT	Self Consistency	Precision	First Run	Third Run	1	No significant difference
Intracranial Hemorrhage	GPT	Self Consistency	Precision	Second Run	Third Run	< 0.0001	Significant difference
Intracranial Hemorrhage	GPT	Self Consistency	Recall	First Run	Second Run	< 0.0001	Significant difference
Intracranial Hemorrhage	GPT	Self Consistency	Recall	First Run	Third Run	0.66	No significant difference
Intracranial Hemorrhage	GPT	Self Consistency	Recall	Second Run	Third Run	< 0.0001	Significant difference
Intracranial Hemorrhage	GPT	Standard	Accuracy	First Run	Second Run	0.94	No significant difference
Intracranial Hemorrhage	GPT	Standard	Accuracy	First Run	Third Run	0.0003	No significant difference
Intracranial Hemorrhage	GPT	Standard	Accuracy	Second Run	Third Run	0.0004	No significant difference
Intracranial Hemorrhage	GPT	Standard	F1 measure	First Run	Second Run	0.33	No significant difference
Intracranial Hemorrhage	GPT	Standard	F1 measure	First Run	Third Run	< 0.0001	Significant difference
Intracranial Hemorrhage	GPT	Standard	F1 measure	Second Run	Third Run	< 0.0001	Significant difference
Intracranial Hemorrhage	GPT	Standard	Precision	First Run	Second Run	< 0.0001	Significant difference
Intracranial Hemorrhage	GPT	Standard	Precision	First Run	Third Run	0.89	No significant difference
Intracranial Hemorrhage	GPT	Standard	Precision	Second Run	Third Run	< 0.0001	Significant difference
Intracranial Hemorrhage	GPT	Standard	Recall	First Run	Second Run	0.19	No significant difference
Intracranial Hemorrhage	GPT	Standard	Recall	First Run	Third Run	< 0.0001	Significant difference
Intracranial Hemorrhage	GPT	Standard	Recall	Second Run	Third Run	< 0.0001	Significant difference
Intracranial Hemorrhage	Gemini	Chain of Thought	Accuracy	First Run	Second Run	< 0.0001	Significant difference
Intracranial Hemorrhage	Gemini	Chain of Thought	Accuracy	First Run	Third Run	< 0.0001	Significant difference
Intracranial Hemorrhage	Gemini	Chain of Thought	Accuracy	Second Run	Third Run	0.46	No significant difference
Intracranial Hemorrhage	Gemini	Chain of Thought	F1 measure	First Run	Second Run	< 0.0001	Significant difference
Intracranial Hemorrhage	Gemini	Chain of Thought	F1 measure	First Run	Third Run	< 0.0001	Significant difference
Intracranial Hemorrhage	Gemini	Chain of Thought	F1 measure	Second Run	Third Run	0.2	No significant difference

Intracranial Hemorrhage	Gemini	Chain of Thought	Precision	First Run	Second Run	< 0.0001	Significant difference
Intracranial Hemorrhage	Gemini	Chain of Thought	Precision	First Run	Third Run	< 0.0001	Significant difference
Intracranial Hemorrhage	Gemini	Chain of Thought	Precision	Second Run	Third Run	0.26	No significant difference
Intracranial Hemorrhage	Gemini	Chain of Thought	Recall	First Run	Second Run	< 0.0001	Significant difference
Intracranial Hemorrhage	Gemini	Chain of Thought	Recall	First Run	Third Run	< 0.0001	Significant difference
Intracranial Hemorrhage	Gemini	Chain of Thought	Recall	Second Run	Third Run	0.87	No significant difference
Intracranial Hemorrhage	Gemini	Self Consistency	Accuracy	First Run	Second Run	< 0.0001	Significant difference
Intracranial Hemorrhage	Gemini	Self Consistency	Accuracy	First Run	Third Run	< 0.0001	Significant difference
Intracranial Hemorrhage	Gemini	Self Consistency	Accuracy	Second Run	Third Run	< 0.0001	Significant difference
Intracranial Hemorrhage	Gemini	Self Consistency	F1 measure	First Run	Second Run	0.0001	Significant difference
Intracranial Hemorrhage	Gemini	Self Consistency	F1 measure	First Run	Third Run	< 0.0001	Significant difference
Intracranial Hemorrhage	Gemini	Self Consistency	F1 measure	Second Run	Third Run	< 0.0001	Significant difference
Intracranial Hemorrhage	Gemini	Self Consistency	Precision	First Run	Second Run	0.001	No significant difference
Intracranial Hemorrhage	Gemini	Self Consistency	Precision	First Run	Third Run	< 0.0001	Significant difference
Intracranial Hemorrhage	Gemini	Self Consistency	Precision	Second Run	Third Run	< 0.0001	Significant difference
Intracranial Hemorrhage	Gemini	Self Consistency	Recall	First Run	Second Run	< 0.0001	Significant difference
Intracranial Hemorrhage	Gemini	Self Consistency	Recall	First Run	Third Run	< 0.0001	Significant difference
Intracranial Hemorrhage	Gemini	Self Consistency	Recall	Second Run	Third Run	0.02	No significant difference
Intracranial Hemorrhage	Gemini	Standard	Accuracy	First Run	Second Run	< 0.0001	Significant difference
Intracranial Hemorrhage	Gemini	Standard	Accuracy	First Run	Third Run	< 0.0001	Significant difference
Intracranial Hemorrhage	Gemini	Standard	Accuracy	Second Run	Third Run	< 0.0001	Significant difference
Intracranial Hemorrhage	Gemini	Standard	F1 measure	First Run	Second Run	< 0.0001	Significant difference
Intracranial Hemorrhage	Gemini	Standard	F1 measure	First Run	Third Run	< 0.0001	Significant difference
Intracranial Hemorrhage	Gemini	Standard	F1 measure	Second Run	Third Run	< 0.0001	Significant difference
Intracranial Hemorrhage	Gemini	Standard	Precision	First Run	Second Run	0.22	No significant difference
Intracranial Hemorrhage	Gemini	Standard	Precision	First Run	Third Run	< 0.0001	Significant difference
Intracranial Hemorrhage	Gemini	Standard	Precision	Second Run	Third Run	< 0.0001	Significant difference
Intracranial Hemorrhage	Gemini	Standard	Recall	First Run	Second Run	< 0.0001	Significant difference
Intracranial Hemorrhage	Gemini	Standard	Recall	First Run	Third Run	< 0.0001	Significant difference
Intracranial Hemorrhage	Gemini	Standard	Recall	Second Run	Third Run	< 0.0001	Significant difference
Skull Fracture	Claude	Chain of Thought	Accuracy	First Run	Second Run	1	No significant difference
Skull Fracture	Claude	Chain of Thought	Accuracy	First Run	Third Run	1	No significant difference
Skull Fracture	Claude	Chain of Thought	Accuracy	Second Run	Third Run	1	No significant difference
Skull Fracture	Claude	Chain of Thought	F1 measure	First Run	Second Run	1	No significant difference
Skull Fracture	Claude	Chain of Thought	F1 measure	First Run	Third Run	1	No significant difference
Skull Fracture	Claude	Chain of Thought	F1 measure	Second Run	Third Run	1	No significant difference
Skull Fracture	Claude	Chain of Thought	Precision	First Run	Second Run	1	No significant difference
Skull Fracture	Claude	Chain of Thought	Precision	First Run	Third Run	1	No significant difference
Skull Fracture	Claude	Chain of Thought	Precision	Second Run	Third Run	1	No significant difference
Skull Fracture	Claude	Chain of Thought	Recall	First Run	Second Run	1	No significant difference
Skull Fracture	Claude	Chain of Thought	Recall	First Run	Third Run	1	No significant difference

Skull Fracture	Claude	Chain of Thought	Recall	Second Run	Third Run	1	No significant difference
Skull Fracture	Claude	Self Consistency	Accuracy	First Run	Second Run	1	No significant difference
Skull Fracture	Claude	Self Consistency	Accuracy	First Run	Third Run	1	No significant difference
Skull Fracture	Claude	Self Consistency	Accuracy	Second Run	Third Run	1	No significant difference
Skull Fracture	Claude	Self Consistency	F1 measure	First Run	Second Run	1	No significant difference
Skull Fracture	Claude	Self Consistency	F1 measure	First Run	Third Run	1	No significant difference
Skull Fracture	Claude	Self Consistency	F1 measure	Second Run	Third Run	1	No significant difference
Skull Fracture	Claude	Self Consistency	Precision	First Run	Second Run	1	No significant difference
Skull Fracture	Claude	Self Consistency	Precision	First Run	Third Run	1	No significant difference
Skull Fracture	Claude	Self Consistency	Precision	Second Run	Third Run	1	No significant difference
Skull Fracture	Claude	Self Consistency	Recall	First Run	Second Run	1	No significant difference
Skull Fracture	Claude	Self Consistency	Recall	First Run	Third Run	1	No significant difference
Skull Fracture	Claude	Self Consistency	Recall	Second Run	Third Run	1	No significant difference
Skull Fracture	Claude	Standard	Accuracy	First Run	Second Run	1	No significant difference
Skull Fracture	Claude	Standard	Accuracy	First Run	Third Run	1	No significant difference
Skull Fracture	Claude	Standard	Accuracy	Second Run	Third Run	1	No significant difference
Skull Fracture	Claude	Standard	F1 measure	First Run	Second Run	1	No significant difference
Skull Fracture	Claude	Standard	F1 measure	First Run	Third Run	1	No significant difference
Skull Fracture	Claude	Standard	F1 measure	Second Run	Third Run	1	No significant difference
Skull Fracture	Claude	Standard	Precision	First Run	Second Run	1	No significant difference
Skull Fracture	Claude	Standard	Precision	First Run	Third Run	1	No significant difference
Skull Fracture	Claude	Standard	Precision	Second Run	Third Run	1	No significant difference
Skull Fracture	Claude	Standard	Recall	First Run	Second Run	1	No significant difference
Skull Fracture	Claude	Standard	Recall	First Run	Third Run	1	No significant difference
Skull Fracture	Claude	Standard	Recall	Second Run	Third Run	1	No significant difference
Skull Fracture	GPT	Chain of Thought	Accuracy	First Run	Second Run	< 0.0001	Significant difference
Skull Fracture	GPT	Chain of Thought	Accuracy	First Run	Third Run	< 0.0001	Significant difference
Skull Fracture	GPT	Chain of Thought	Accuracy	Second Run	Third Run	0.82	No significant difference
Skull Fracture	GPT	Chain of Thought	F1 measure	First Run	Second Run	0.2	No significant difference
Skull Fracture	GPT	Chain of Thought	F1 measure	First Run	Third Run	< 0.0001	Significant difference
Skull Fracture	GPT	Chain of Thought	F1 measure	Second Run	Third Run	0.00024	No significant difference
Skull Fracture	GPT	Chain of Thought	Precision	First Run	Second Run	< 0.0001	Significant difference
Skull Fracture	GPT	Chain of Thought	Precision	First Run	Third Run	0.85	No significant difference
Skull Fracture	GPT	Chain of Thought	Precision	Second Run	Third Run	< 0.0001	Significant difference
Skull Fracture	GPT	Chain of Thought	Recall	First Run	Second Run	0.04	No significant difference
Skull Fracture	GPT	Chain of Thought	Recall	First Run	Third Run	< 0.0001	Significant difference
Skull Fracture	GPT	Chain of Thought	Recall	Second Run	Third Run	< 0.0001	Significant difference
Skull Fracture	GPT	Self Consistency	Accuracy	First Run	Second Run	0.03	No significant difference
Skull Fracture	GPT	Self Consistency	Accuracy	First Run	Third Run	< 0.0001	Significant difference
Skull Fracture	GPT	Self Consistency	Accuracy	Second Run	Third Run	0.0001	Significant difference
Skull Fracture	GPT	Self Consistency	F1 measure	First Run	Second Run	0.03	No significant difference

Skull Fracture	GPT	Self Consistency	F1 measure	First Run	Third Run	< 0.0001	Significant difference
Skull Fracture	GPT	Self Consistency	F1 measure	Second Run	Third Run	< 0.0001	Significant difference
Skull Fracture	GPT	Self Consistency	Precision	First Run	Second Run	0.002	No significant difference
Skull Fracture	GPT	Self Consistency	Precision	First Run	Third Run	0.82	No significant difference
Skull Fracture	GPT	Self Consistency	Precision	Second Run	Third Run	0.001	No significant difference
Skull Fracture	GPT	Self Consistency	Recall	First Run	Second Run	1	No significant difference
Skull Fracture	GPT	Self Consistency	Recall	First Run	Third Run	< 0.0001	Significant difference
Skull Fracture	GPT	Self Consistency	Recall	Second Run	Third Run	< 0.0001	Significant difference
Skull Fracture	GPT	Standard	Accuracy	First Run	Second Run	0.22	No significant difference
Skull Fracture	GPT	Standard	Accuracy	First Run	Third Run	< 0.0001	Significant difference
Skull Fracture	GPT	Standard	Accuracy	Second Run	Third Run	< 0.0001	Significant difference
Skull Fracture	GPT	Standard	F1 measure	First Run	Second Run	0.0002	No significant difference
Skull Fracture	GPT	Standard	F1 measure	First Run	Third Run	< 0.0001	Significant difference
Skull Fracture	GPT	Standard	F1 measure	Second Run	Third Run	< 0.0001	Significant difference
Skull Fracture	GPT	Standard	Precision	First Run	Second Run	0.91	No significant difference
Skull Fracture	GPT	Standard	Precision	First Run	Third Run	< 0.0001	Significant difference
Skull Fracture	GPT	Standard	Precision	Second Run	Third Run	< 0.0001	Significant difference
Skull Fracture	GPT	Standard	Recall	First Run	Second Run	0.48	No significant difference
Skull Fracture	GPT	Standard	Recall	First Run	Third Run	< 0.0001	Significant difference
Skull Fracture	GPT	Standard	Recall	Second Run	Third Run	< 0.0001	Significant difference
Skull Fracture	Gemini	Chain of Thought	Accuracy	First Run	Second Run	< 0.0001	Significant difference
Skull Fracture	Gemini	Chain of Thought	Accuracy	First Run	Third Run	< 0.0001	Significant difference
Skull Fracture	Gemini	Chain of Thought	Accuracy	Second Run	Third Run	0.004	No significant difference
Skull Fracture	Gemini	Chain of Thought	F1 measure	First Run	Second Run	< 0.0001	Significant difference
Skull Fracture	Gemini	Chain of Thought	F1 measure	First Run	Third Run	< 0.0001	Significant difference
Skull Fracture	Gemini	Chain of Thought	F1 measure	Second Run	Third Run	0.01	No significant difference
Skull Fracture	Gemini	Chain of Thought	Precision	First Run	Second Run	< 0.0001	Significant difference
Skull Fracture	Gemini	Chain of Thought	Precision	First Run	Third Run	< 0.0001	Significant difference
Skull Fracture	Gemini	Chain of Thought	Precision	Second Run	Third Run	0.001	No significant difference
Skull Fracture	Gemini	Chain of Thought	Recall	First Run	Second Run	0.03	No significant difference
Skull Fracture	Gemini	Chain of Thought	Recall	First Run	Third Run	0.03	No significant difference
Skull Fracture	Gemini	Chain of Thought	Recall	Second Run	Third Run	1	No significant difference
Skull Fracture	Gemini	Self Consistency	Accuracy	First Run	Second Run	0.82	No significant difference
Skull Fracture	Gemini	Self Consistency	Accuracy	First Run	Third Run	< 0.0001	Significant difference
Skull Fracture	Gemini	Self Consistency	Accuracy	Second Run	Third Run	< 0.0001	Significant difference
Skull Fracture	Gemini	Self Consistency	F1 measure	First Run	Second Run	0.9	No significant difference
Skull Fracture	Gemini	Self Consistency	F1 measure	First Run	Third Run	< 0.0001	Significant difference
Skull Fracture	Gemini	Self Consistency	F1 measure	Second Run	Third Run	< 0.0001	Significant difference
Skull Fracture	Gemini	Self Consistency	Precision	First Run	Second Run	0.89	No significant difference
Skull Fracture	Gemini	Self Consistency	Precision	First Run	Third Run	< 0.0001	Significant difference
Skull Fracture	Gemini	Self Consistency	Precision	Second Run	Third Run	< 0.0001	Significant difference

Skull Fracture	Gemini	Self Consistency	Recall	First Run	Second Run	1	No significant difference
Skull Fracture	Gemini	Self Consistency	Recall	First Run	Third Run	< 0.0001	Significant difference
Skull Fracture	Gemini	Self Consistency	Recall	Second Run	Third Run	< 0.0001	Significant difference
Skull Fracture	Gemini	Standard	Accuracy	First Run	Second Run	0.01	No significant difference
Skull Fracture	Gemini	Standard	Accuracy	First Run	Third Run	< 0.0001	Significant difference
Skull Fracture	Gemini	Standard	Accuracy	Second Run	Third Run	< 0.0001	Significant difference
Skull Fracture	Gemini	Standard	F1 measure	First Run	Second Run	0.2	No significant difference
Skull Fracture	Gemini	Standard	F1 measure	First Run	Third Run	< 0.0001	Significant difference
Skull Fracture	Gemini	Standard	F1 measure	Second Run	Third Run	< 0.0001	Significant difference
Skull Fracture	Gemini	Standard	Precision	First Run	Second Run	0.04	No significant difference
Skull Fracture	Gemini	Standard	Precision	First Run	Third Run	< 0.0001	Significant difference
Skull Fracture	Gemini	Standard	Precision	Second Run	Third Run	< 0.0001	Significant difference
Skull Fracture	Gemini	Standard	Recall	First Run	Second Run	1	No significant difference
Skull Fracture	Gemini	Standard	Recall	First Run	Third Run	< 0.0001	Significant difference
Skull Fracture	Gemini	Standard	Recall	Second Run	Third Run	< 0.0001	Significant difference

LLM large language model

Supplementary Table 3: Error analyses of large language models' structured reporting by institution

Label	Expected cause of incorrect interpretation	Institution A	Institution B	Institution C	Total (case)
Hemorrhage	Contains expressions such as possible/cannot be ruled out/suspected/cannot be identified	55	7	9	71
	Incorrect interpretation despite clear hemorrhage description	35	12	16	63
	Contains descriptions of unchanged hemorrhage	2	40	16	58
	Contains descriptions of edema/swelling/hypodense area/cerebral infarction	4	3	32	39
	Contains descriptions of hemorrhage reduction/improvement/decreased density/becoming unclear	4	25	9	38
	Contains descriptions of extracranial hemorrhage	30	1	4	35
	Contains descriptions of intracranial mass	7	2	6	15
	Contains descriptions of post-hemorrhagic scarring	3	0	1	4
	Contains descriptions of pseudo-subarachnoid hemorrhage	0	3	0	3
	Contains descriptions of thrombosis/hyperdense sign	0	0	2	2
	Contains descriptions of post-hemorrhage removal status	0	1	1	2
	Contains descriptions of fractures	2	0	0	2
	Contains descriptions of contrast extravasation	0	0	1	1
	Contains descriptions of laminar necrosis	1	0	0	1
Fracture	Incorrect interpretation despite clear fracture description	8	1	1	10
	Contains expressions such as possible/cannot be ruled out/suspected/cannot be identified	8	1	0	9
	Contains descriptions of unclear fracture	0	1	0	1
	Contains descriptions of post-fracture surgery status	1	1	1	3
	Contains descriptions of post-craniotomy changes	8	4	9	21
	Contains descriptions of unchanged fracture	0	2	3	5
	Contains descriptions of cervical vertebra fracture	1	3	4	8
	Contains descriptions of nasal septum deviation/nasal bone deformity	2	0	1	3
	Contains descriptions of old fracture	3	0	0	3
	Contains descriptions of contusion/hemorrhage	2	1	5	8
	Contains descriptions of bone defect/destruction	3	4	1	8
	Contains descriptions of bone mass	3	0	1	4
	Contains descriptions of basilar invagination	0	1	0	1
	Contains descriptions of soft tissue density in paranasal sinuses/temporal bone	1	0	0	1

Expected cause of incorrect interpretation may contain duplicates
